# Prediction of drug cocktail effects when the number of measurements is limited

**DOI:** 10.1371/journal.pbio.2002518

**Published:** 2017-10-26

**Authors:** Anat Zimmer, Avichai Tendler, Itay Katzir, Avi Mayo, Uri Alon

**Affiliations:** Department of Molecular Cell Biology, Weizmann Institute of Science, Rehovot, Israel; Institute for Systems Biology, United States of America

## Abstract

Cocktails of drugs can be more effective than single drugs, because they can potentially work at lower doses and avoid resistance. However, it is impossible to test all drug cocktails drawn from a large set of drugs because of the huge number of combinations. To overcome this combinatorial explosion problem, one can sample a relatively small number of combinations and use a model to predict the rest. Recently, Zimmer and Katzir et al. presented a model that accurately predicted the effects of cocktails at all doses based on measuring pairs of drugs. This model requires measuring each pair at several different doses and uses interpolation to reduce experimental noise. However, often, it is not possible to measure each pair at multiple doses (for example, in scarce patient-derived tumor material or in large screens). Here, we ask whether measurements at only a single dose can also predict high-order drug cocktails. To address this, we present a fully factorial experimental dataset on all drug cocktails built of 6 chemotherapy drugs on 2 cancer cell lines. We develop a formula that uses only pair measurements at a single dose to predict much of the variation up to 6-drug cocktails in the present data, outperforming commonly used Bliss independence and regression approaches. This model, called the pairs model, is an extension of the Bliss independence model to pairs: For M drugs, it equals the product of all pair effects to the power 1/(M−1). The pairs model also shows good agreement with previously published data on antibiotic triplets and quadruplets. The present model can only predict combinations at the same doses in which the pairs were measured and is not able to predict effects at other doses. This study indicates that pair-based approaches might be able to usefully predict and prioritize high-order combinations, even in large screens or when material for testing is limited.

## Author summary

Drug cocktails are a promising strategy for diseases such as cancer and infections, because cocktails can be more effective than individual drugs and can overcome problems of drug resistance. However, finding the best cocktail comprising a given set of drugs is challenging because the number of experiments needed is huge and grows exponentially with the number of drugs. This problem is exacerbated when the experiments are expensive and the material for testing is rare. Here, we present a way to address this challenge using a mathematical formula, called the pairs model, that requires relatively few experimental tests in order to estimate the effects of cocktails and to predict which cocktail is most effective. The formula does well on experimental data generated in this study using combinations of between 3 and 6 anticancer drugs, as well as on existing data that use combinations of antibiotics.

## Introduction

Cancer and antibiotic treatments face the problem of drug resistance. Cancer drugs also face problems of efficacy at the low doses needed to tolerate side effects [1–5]. One strategy to overcome these challenges is cocktails of several drugs [5–9]. Cocktails overcame the challenges of resistance and efficacy in diseases such as HIV [10–12] and are used in diverse medical contexts. Effective cocktails can, in principle, be designed for each individual patient [13].

Currently, there are approximately 1,000 available compounds to treat cancer [14,15]. Testing all combinations at all doses is impossible, because the number of experiments grows exponentially with the number of drugs and doses. Hence, very effective cocktails may be hidden in this vast space of possible combinations [1,6,16,17], as recently demonstrated by Horn et al. in an extensive study of colorectal cancer [5].

Current approaches to overcome the combinatorial explosion problem of drug combinations use mathematical models to predict the effects of combinations based on a small number of measurements [18–20]. The commonly used Bliss model is a reasonable first approximation but does not include synergy and antagonism effects. Machine learning approaches have been proposed, but the large datasets needed to train these models are still not widely available [21–26]. The Isserlis model proposed by Wood et al. showed excellent results on antibiotic combinations of 3 and 4 drugs [27–29]. Additional studies considered the interactions of multiple drugs using first- and second-order terms for drug effects [30–38].

Recently, Zimmer and Katzir et al. suggested a model that accurately predicted the multidose response of triplets and quadruplets of antibiotics and cancer drugs [29]. This model requires measurements of each pair of drugs at a few dose combinations. It uses this multidose information to fit a smooth drug response curve for all doses, in which each drug changes the effective dose of the other drugs. This model greatly reduces the number of experiments needed in order to scan the space of drug cocktails at multiple doses.

Data at multiple doses required for the multidose model of [29] is not always available. This fact is most pressing when the measurements are made on rare materials whose limited quantity allows only a few tests, such as patient-derived material in individualized medicine applications [39–42] or in expensive high-throughput screens [43–45]. There is therefore need for a model that uses measurements at a single dose in order to predict the effects of high-order combinations (such a model is expected to work only at the measured doses and no other doses). In particular, previous models were tested only up to triplets and quadruplets of drugs. Experiments on higher-order combinations are hence of interest [5].

To address this, we tested all combinations of 6 chemotherapy drugs at a single dose on 2 cell lines (a fully factorial design). We find that synergy and antagonism are cell-line dependent and are usually consistent with the synergy/antagonism of the pairs that make up each combination. We developed a simple model for cocktails that is insensitive to experimental noise and that uses only measurements on drug pairs at a single dose. In addition to the 6-drug combinations, we further tested the model on previous fully-factorial datasets of 3 chemotherapy drugs at 8 doses [29] and of 3 or 4 antibiotics [28], totaling 1,392 additional triplets and 248 quadruplets. The pairs model predicts well the effect of these cocktails, with the limitation that it can only predict effects at the same dose at which the pairs were measured.

## Results

### Fully factorial experiments on 6 drugs and 2 cell lines

We selected 6 cytotoxic drugs from several clinically employed drug families with different mechanisms of action (Table 1). We chose drugs that are used in combination in some clinical settings, such as an alkylating agent with a microtubule poison. For each drug, we measured cell survival after 48 h using the neutral red assay. A second survival assay, MTT, gave very similar results (S1 Fig). Each measurement was repeated on at least 6 biological repeats, in triplicate.

**Table 1:** Cytotoxic drugs and their mechanisms of action, LD20 concentrations, and Hill coefficients. LD20 concentrations are the drug concentrations in μM that kill 20% of the cells, and *n* is the Hill coefficient of the dose response curves.

				H1299		HeLa	
Drug name	Abbreviation	Target	Target and damage mode	LD20 [uM]	*n*	LD20 [uM]	*n*
Camptothecin	CPT	Topoisomerase I	Topoisomerase I poison, that binds and stabilizes the topoisomerase I-DNA complex, causing DNA damage and cell death.	0.1	0.5	0.15	0.3
Cisplatinum	CisPt	DNA	Crosslinks DNA in several different ways, making it impossible for rapidly dividing cells to duplicate their DNA for mitosis and leading to DNA damage and cell death.	13.8	2.2	7	2.1
Nocodazole	NCZ	Tubulin	An antimitotic drug that binds tubulin at high affinity and inhibits microtubule assembly thus promoting microtubule depolymerization.	50	0.6	0.01	0.95
Etoposide	Etopo	Topoisomerase II	Binds to and inhibits topoisomerase II, resulting in the accumulation of single- or double-strand DNA breaks. This results in the inhibition of DNA replication and transcription and apoptotic cell death.	10	0.85	6	0.5
Carboplatin	CbPT	DNA	Thought to act similarly to cisplatinum though with reduced side effects.	200	2.2	165	1.6
Proteasome inhibitor	MG132	Proteasome	Proteasome inhibitor.	0.15	1.5	0.38	1.2

**Abbreviations:** CbPt, Carboplatin; CisPt, Cisplatin; CPT, Camptothecin; Etopo, Etoposide; LD20, lethal dose 20%; NCZ, Nocodazole

We repeated this for 2 human cancer cell lines from different cancers and genetic backgrounds, H1299 and HeLa. The non-small-cell lung carcinoma line H1299 was derived from metastatic lymph node from a male patient and characterized by partial homozygous deletion in the p53 gene, resulting in lack of expression of the p53 protein [46]. HeLa is a cervical cancer cell line. HeLa cells express p53 at low levels and were derived from a primary tumor from a female patient [47].

We first measured single-drug dose response curves, which were well described by Hill functions with moderate cooperativity (Table 1). For each drug, we chose a dose that shows about 80% survival, lethal dose 20% (LD20; we chose a dose of high survival in order to have sensitive measurements when combining 6 of the drugs). We then measured all 63 combinations of the six drugs: 6 singles, 15 pairs, 20 triplets, 15 quadruplets, 6 quintuplets, and the sextuplet. In the case of HeLa cells, 17 of the combinations had large variations between repeats and were not included. The results for all measured drug combinations are shown in Table 2 (and S1 Table), as well as interactions and their confidence intervals based on bootstrapping the biological repeats.

**Table 2:** Cell survival and interactions of all drug combinations in the present study. Viability was measured using the neutral read assay. 5% CI and 95% CI mark 5% and 95% confidence intervals from bootstrapping. Interaction is I = log(1 + viability − Bliss) where Bliss is the product of the single-drug viability values. Antagonism and synergy are marked in blue and pink and denote cases where I > 0 or I < 0 together with the demand that I = 0 is outside the 95% confidence interval. Cocktails in bold are synergistic/antagonistic for both cell lines.

		H1299					HeLa				
	Drug cocktail	viability	ste	Interaction	5% CI	95% CI	viability	ste	Interaction	5% CI	95% CI
Singles	CPT	0.77	0.01				0.73	0.02			
	CisPt	0.82	0.01				0.65	0.03			
	CbPt	0.77	0.01				0.27	0.04			
	NCZ	0.73	0.02				0.83	0.02			
	MG132	0.68	0.02				0.70	0.02			
	Etopo	0.72	0.01				0.71	0.02			
Pairs	**CPT+CisPt**	0.67	0.02	0.04	0.01	0.08	0.62	0.02	0.13	0.10	0.16
	CPT+CbPt	0.58	0.02	−0.01	−0.05	0.02	0.09	0.01	−0.14	−0.19	−0.09
	**CPT+NCZ**	0.66	0.02	0.07	0.04	0.09	0.66	0.03	0.10	0.07	0.13
	**CPT+MG132**	0.66	0.01	0.12	0.09	0.15	0.76	0.02	0.21	0.17	0.24
	CPT+Etopo	0.51	0.01	−0.05	−0.08	−0.02	0.60	0.02	0.07	0.04	0.10
	CisPt+CbPt	0.73	0.02	0.08	0.05	0.11	0.11	0.02	−0.07	−0.09	−0.05
	**CisPt+NCZ**	0.72	0.02	0.10	0.07	0.12	0.87	0.04	0.24	0.19	0.29
	**CisPt+MG132**	0.74	0.02	0.15	0.12	0.18	0.80	0.03	0.28	0.24	0.32
	**CisPt+Etopo**	0.68	0.02	0.08	0.06	0.11	0.63	0.01	0.15	0.11	0.18
	CbPt+NCZ	0.41	0.02	−0.17	−0.23	−0.11	-	-	-	-	-
	**CbPt+MG132**	0.58	0.02	0.06	0.03	0.09	0.25	0.03	0.05	0.03	0.07
	CbPt+Etopo	0.64	0.02	0.08	0.06	0.11	0.17	0.01	−0.02	−0.06	0.01
	NCZ+MG132	0.47	0.02	−0.04	−0.08	−0.01	0.78	0.02	0.15	0.11	0.19
	**NCZ+Etopo**	0.78	0.02	0.23	0.20	0.25	0.77	0.03	0.18	0.13	0.22
	**MG132+Etopo**	0.72	0.02	0.21	0.18	0.24	0.83	0.02	0.28	0.23	0.32
Triplets	**CPT+CisPt+CbPt**	0.37	0.04	−0.07	−0.13	−0.01	0.08	0.00	−0.07	−0.10	−0.04
	CPT+CisPt+NCZ	0.31	0.03	−0.16	-0.26	−0.07	0.60	0.02	0.19	0.13	0.23
	CPT+CisPt+MG132	0.37	0.01	−0.04	−0.10	0.02	0.55	0.01	0.18	0.14	0.22
	CPT+CisPt+Etopo	0.40	0.04	−0.03	−0.12	0.07	0.41	0.01	0.06	0.03	0.09
	CPT+CbPt+NCZ	0.53	0.01	0.05	−0.01	0.12	-	-	-	-	-
	**CPT+CbPt+MG132**	0.31	0.02	−0.06	−0.13	0.00	0.08	0.04	−0.07	−0.09	−0.05
	CPT+CbPt+Etopo	0.32	0.03	−0.08	−0.20	0.04	0.02	0.00	−0.14	−0.19	−0.10
	CPT+NCZ+MG132	0.28	0.05	−0.11	−0.18	−0.05	0.63	0.02	0.19	0.17	0.22
	CPT+NCZ+Etopo	0.34	0.00	−0.08	−0.14	−0.02	0.54	0.00	0.14	0.10	0.19
	**CPT+MG132+Etopo**	0.58	0.03	0.14	0.05	0.25	0.64	0.01	0.22	0.16	0.27
	CisPt+CbPt+NCZ	0.18	0.01	−0.35	−0.45	−0.26	-	-	-	-	-
	CisPt+CbPt+MG132	0.55	0.01	0.08	−0.02	0.17	0.10	0.00	−0.04	−0.06	−0.01
	CisPt+CbPt+Etopo	0.52	0.02	0.02	−0.03	0.07	0.07	0.01	−0.07	−0.09	−0.04
	CisPt+NCZ+MG132	0.22	0.01	−0.25	−0.42	−0.10	0.74	0.02	0.26	0.23	0.30
	**CisPt+NCZ+Etopo**	0.68	0.01	0.20	0.15	0.25	0.64	0.01	0.20	0.17	0.24
	**CisPt+MG132+Etopo**	0.72	0.01	0.26	0.21	0.31	0.61	0.01	0.24	0.21	0.27
	CbPt+NCZ+MG132	0.15	0.01	−0.30	−0.51	−0.14	-	-	-	-	-
	CbPt+NCZ+Etopo	0.29	0.01	−0.05	−0.12	0.02	-	-	-	-	-
	**CbPt+MG132+Etopo**	0.50	0.01	0.17	0.12	0.23	0.19	0.00	0.05	0.02	0.07
	NCZ+MG132+Etopo	0.30	0.01	0.02	−0.01	0.05	0.71	0.02	0.25	0.20	0.30
Quadruplets	CPT+CisPt+CbPt+NCZ	0.14	0.00	−0.15	−0.21	−0.10	-	-	-	-	-
	CPT+CisPt+CbPt+MG132	0.19	0.01	−0.07	v0.14	0.00	0.03	0.00	−0.09	−0.13	−0.05
	**CPT+CisPt+CbPt+Etopo**	0.10	0.02	−0.22	−0.28	−0.15	0.02	0.00	−0.09	−0.11	−0.06
	CPT+CisPt+NCZ+MG132	0.17	0.01	−0.06	−0.11	−0.01	0.48	0.02	0.17	0.14	0.20
	CPT+CisPt+NCZ+Etopo	0.15	0.01	−0.13	−0.17	−0.09	0.41	0.01	0.12	0.09	0.16
	CPT+CisPt+MG132+Etopo	0.16	0.01	−0.09	−0.15	−0.02	-	-	-	-	-
	CPT+CbPt+NCZ+MG132	0.16	0.00	−0.14	−0.23	−0.07	-	-	-	-	-
	CPT+CbPt+NCZ+Etopo	0.14	0.01	−0.22	−0.28	−0.15	-	-	-	-	-
	CPT+CbPt+MG132+Etopo	0.25	0.01	−0.03	−0.09	0.03	0.03	0.00	−0.06	−0.09	−0.03
	CPT+NCZ+MG132+Etopo	0.21	0.01	−0.08	−0.12	−0.04	0.77	0.02	0.40	0.37	0.42
	CisPt+CbPt+NCZ+MG132	0.10	0.00	−0.24	−0.32	−0.16	-	-	-	-	-
	CisPt+CbPt+NCZ+Etopo	0.21	0.01	−0.10	−0.17	−0.03	-	-	-	-	-
	CisPt+CbPt+MG132+Etopo	0.55	0.01	0.22	0.19	0.26	0.04	0.01	−0.06	−0.09	−0.04
	**CisPt+NCZ+MG132+Etopo**	0.37	0.01	0.08	0.03	0.13	0.80	0.01	0.40	0.37	0.43
	CbPt+NCZ+MG132+Etopo	0.11	0.01	−0.20	−0.25	−0.14	-	-	-	-	-
Quintuplets	CPT+CisPt+CbPt+NCZ+MG132	0.22	0.04	−0.19	−0.27	−0.11	-	-	-	-	-
	CPT+CisPt+CbPt+NCZ+Etopo	0.27	0.02	0.00	−0.06	0.05	-	-	-	-	-
	CPT+CisPt+CbPt+MG132+Etopo	0.43	0.01	0.06	0.00	0.13	0.00	0.00	−0.06	−0.08	−0.04
	CPT+CisPt+NCZ+MG132+Etopo	0.26	0.01	0.01	−0.06	0.08	0.58	0.01	0.32	0.30	0.34
	CPT+CbPt+NCZ+MG132+Etopo	0.17	0.03	−0.15	−0.25	−0.06	-	-	-	-	-
	CisPt+CbPt+NCZ+MG132+Etopo	0.20	0.01	−0.09	−0.14	−0.03	-	-	-	-	-
Sextuplet	CPT+CisPt+CbPt+NCZ+MG132+Etopo	0.29	0.03	0.04	−0.01	0.09	-	-	-	-	-

**Abbreviations:** CbPt, Carboplatin; CisPt, Cisplatin; CPT, Camptothecin; Etopo, Etoposide; LD20, NCZ, Nocodazole

### Some combinations showed synergism in both cell lines, and others were cell-line specific

We measured the interactions between the drugs by comparing the effects of the combination to Bliss independence. The effect of drug i, g_i_, is the fractional reduction in cell survival. In other contexts, g_i_ is the fractional reduction in cell growth rate [28,29,48] or other measures of drug impact. Bliss independence is a model in which the combination effect is the product of the effects of each individual drug

$$g^{\text{Bliss}}{}_{1..\text{k}}={\text{g}}_{1}{\text{g}}_{2}\ldots g_{\text{k}}$$ (1)

We quantified the interaction by the logarithmic deviation from the Bliss model, I = log(1+g_1..k_ − g^Bliss^_1..k_) [49] (for other approaches to calculate synergy, see [5]). Negative numbers indicate synergy, where drugs kill more cells than Bliss independence, and positive numbers indicate antagonism. We computed 95% confidence intervals for each interaction term by bootstrapping over the biological repeats of each measurement. We consider interaction terms as non-zero when I = 0 is not included in their 95% confidence interval.

We find that 30% of the combinations are synergistic for HeLa cells and 44% for H1299 (S2 Fig). The most synergistic combination for H1299 was Cisplatin, Carboplatin, and Nocodazole (CisPt+CbPt+NCZ), with I = −0.36. For HeLa, the most synergistic combination was Camptothecin, Carboplatin, and Etoposide (CPT+CbPt+Etopo) with I = −0.14.

**Fig 1:**
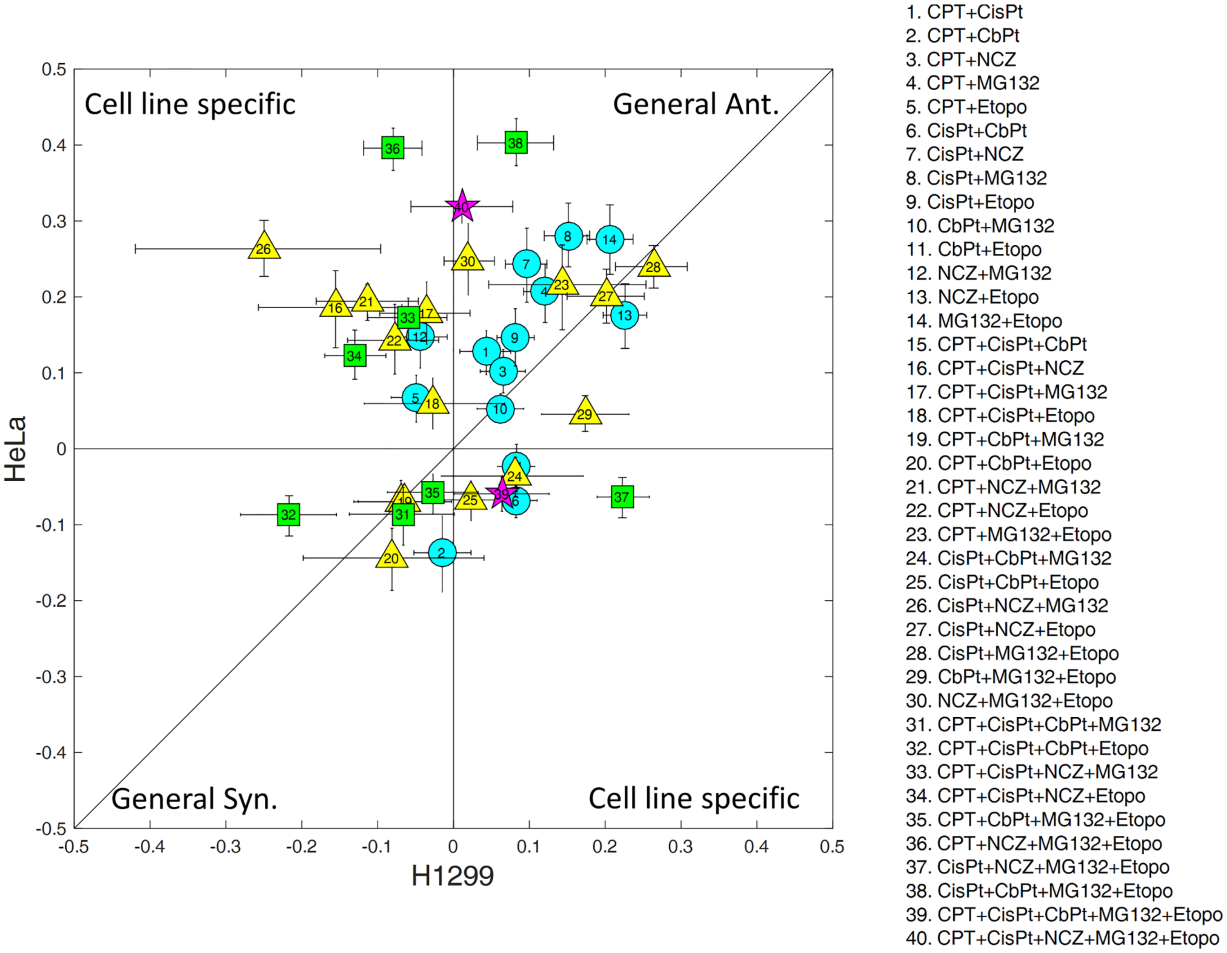
A comparison of drug interactions between the 2 cell lines. Circles stand for cocktails of pairs of drugs, triangles for triplets, squares for quadruplets, and stars for cocktails of 5 drugs. The error bars are 95% confidence intervals of the measurements. The number in each shape identifies the cocktail according to the list on the right. CbPt, Carboplatin; CisPt, Cisplatin; CPT, Camptothecin; Etopo, Etoposide; NCZ, Nocodazole.

We compared the synergy/antagonism of each combination between the 2 cell lines. Fig 1 (and S1 Table) shows each combination plotted by its interaction in HeLa versus H1299. Overall, there is only a moderate correspondence between the interactions in the 2 cell lines (R = 0.16). The higher the combination order, the smaller the correlation between the two cell lines: Two-drug interactions (circles) tend to be more similar between the 2 cell lines (R = 0.5) than triplets (triangles, R = 0.1) and quadruplets (squares, R = 0.08). This indicates that cell-line-specific predictions are needed, especially for the high-order cocktails in this sample, although care is needed with this interpretation due to small number effects and noise.

### The synergy/antagonism sign of a combination in a cell line is often consistent with that of its constituent pairs

**Fig 2:**
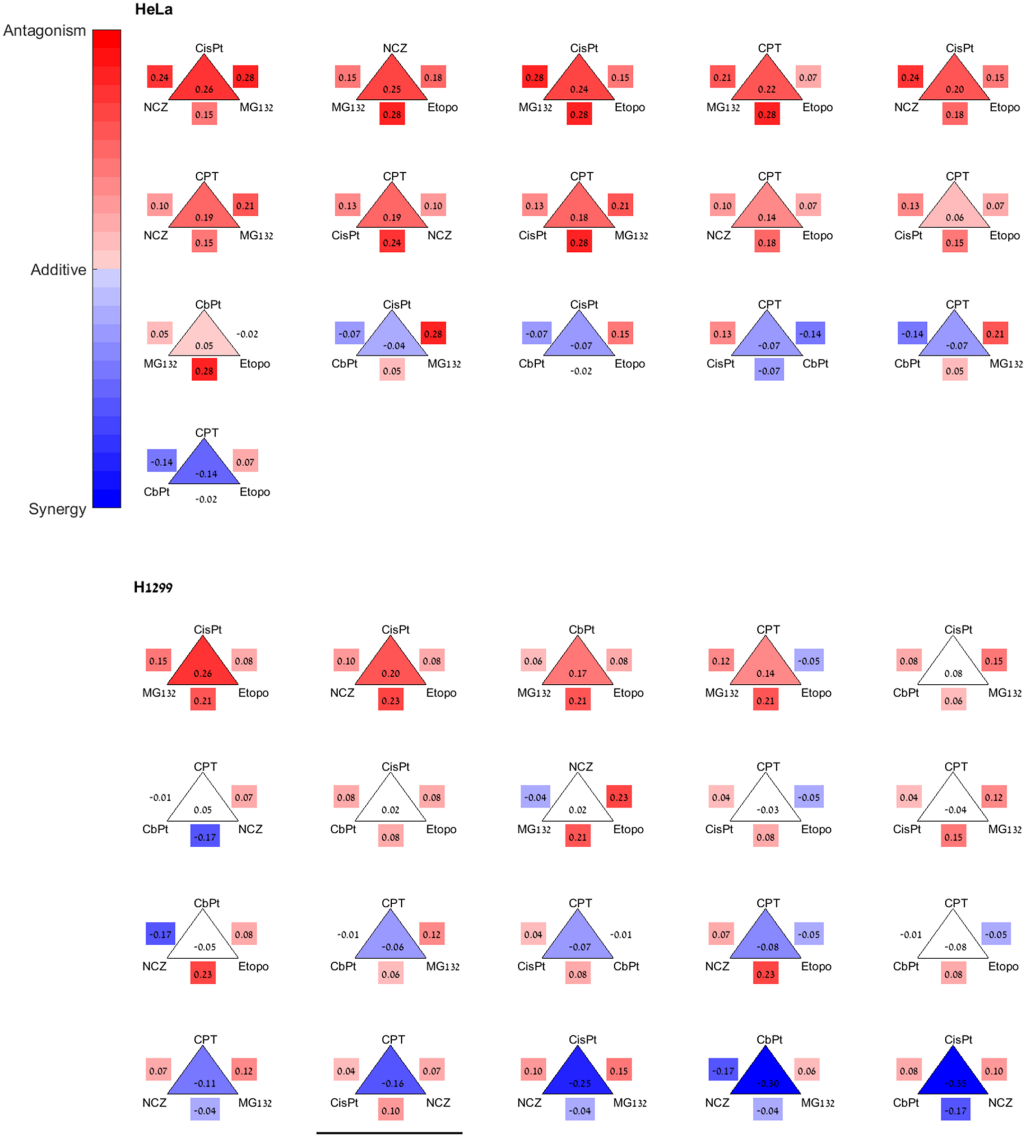
The synergy/antagonism sign of triplets is mostly consistent with that of their constituent pairs. Shown are all triplets of HeLa (upper panel) and H1299 (lower panel). Drug names are at the vertices of the triangles. The number inside the triangle is the triplet interaction (I_123_ = log(1 + g_123_ − g_1_g_2_g_3_)), and edges are marked by the pair interactions. Color indicates the interaction sign: red is antagonistic, blue is synergistic, and white is additive (that is, I = 0 is included in the 95% confidence interval). The triangles are ordered from the most antagonistic triplet to the most synergistic one. The underlined H1299 triplet is the only triplet where the sign of I_123_ is not consistent with the sign of the 3 pair interactions, a possible indication of third-order interaction. Two other synergistic H1299 triplets are marginally inconsistent in the sense that they contain 2 antagonistic pairs and 1 pair that is judged additive according to the present experimental variation. I, interaction. CbPt, Carboplatin; CisPt, Cisplatin; CPT, Camptothecin; Etopo, Etoposide; NCZ, Nocodazole.

To address the possibility of using pairs to model the cocktails, we asked whether the synergy/antagonism sign of pairs in a combination is informative with regards to the overall synergy/antagonism sign of the cocktail in a given cell line. For this purpose, we compared the interaction (I) values of each combination of drugs to the interaction of its constituent pairs. The results for triplets are shown in Fig 2 (and S1 Table).

An indication of high-order interactions is when the signs of the pair interactions do not correspond to the interaction sign of the combination. Such high-order interactions would make it difficult to use pairs to model higher-order combinations, especially if the interaction sign of all pairs is opposite to that of the combination. We find that for HeLa cells all of the cocktails (of 3–6 drugs) that are antagonistic had at least 1 antagonistic pair (16/16), and all the synergistic cocktails had at least 1 synergistic pair (10/10). For H1299, all of the antagonistic cocktails had at least 1 antagonistic pair (7/7), and all but 3 of the synergistic combinations had at least 1 synergistic pair (19/22). The 3 exceptions are all triplets, underlined in Fig 2, and may be candidates for exploring third-order interactions. Taking all data together, the synergy/antagonism sign of the combinations is different from all of the pairs only rarely (6% overall) in this sample, suggesting that drug pairs may carry useful information about the cocktail effects [5,28,29].

### We scanned a family of mathematical models to predict high-order cocktails based on pairs

We next asked to what extent data on drug pairs can improve on the Bliss formula to predict the effect of cocktails. This requires mathematical formula to properly utilize pair data. There are several existing approaches for such pair-based predictions. A widely used machine learning approach is based on logarithmic regression [21–26]. For triplets, regression yields g^Regression^_123_ = g_12_g_23_g_13_/g_1_g_2_g_3_, and for quadruplets $g^{Regression}{}_{1234}=\prod g_{ij}/\prod g_{i}{}^{2}$ [29].

A second approach employs the Isserlis formula introduced by Wood et al. based on maximum entropy considerations [28]. For triplets, the Isserlis formula is g^Isserlis^_123_ = g_1_g_23_ + g_2_g_13_+g_3_g_12_ − 2g_1_g_2_g_3_, and for quadruplets, g^Isserlis^_1234_ = g_12_g_34_ + g_13_g_24_ + g_14_g_23_ − 2g_1_g_2_g_3_g_4_. The Isserlis formula showed excellent agreement with data on antibiotic triplets and quadruplets [28]. The regression and Isserlis formulas have the desirable mathematical property that if all pairs are Bliss (that is, if for all pairs g_ij_ = g_i_ g_j_), then the predicted cocktail is also Bliss g_123..k_ = g_1_g_2_g_3 …_g_k_. We call this property Bliss conservation.

We sought to test additional models that use single drugs and drug pairs as inputs, measured at a single dose. We therefore sought a family of models that generalize the Bliss and regression formulas. We choose the family of log-linear combinations of single and pair measurements, which smoothly interpolates between Bliss and regression. For triplets, we tested g_123_ = (g_12_g_23_g_13_)^α^(g_1_g_2_g_3_)^β^, where α and β are parameters. To preserve the Bliss conservation property requires β = 1 − 2α. Generalizing to M drugs, we have $g_{1\ldots M}={\left(\prod g_{ij}\right)}^{\alpha }{\left(\prod g_{i}\right)}^{\beta }$ with β = 1 − (M − 1)α. The Bliss formula is obtained when α = 0 and the regression formula when α = 1.

**Fig 3:**
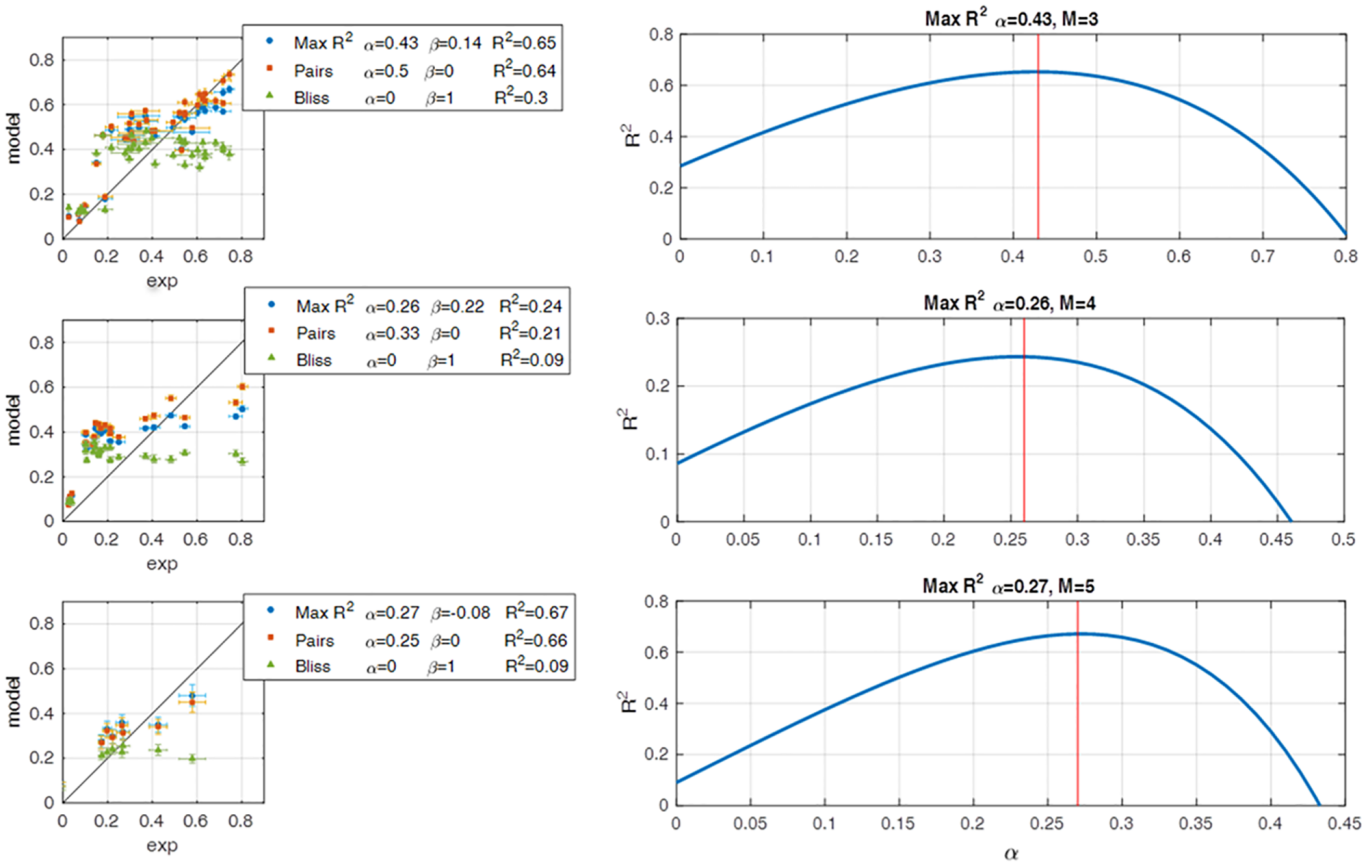
To define different models, we scanned the log-linear model family over α while setting β = 1 − (M − 1)α and calculated the R^2^ values. The maximal R^2^ occurs close to α = 1/(M − 1) and β = 0, which is the definition of the pairs model. The left panel is a comparison between the pairs model, the Bliss model, and the model defined by the α with the highest R^2^. The left panel is the R^2^ values of α = 0:0.05:1. The rows are for M = 3, 4, and 5, respectively.

We scanned the value of the parameter α, setting β = 1 − (M − 1)α to preserve Bliss conservation, and compared the R^2^ values for the fully factorial datasets described above. We find that a simple formula shows nearly maximal R^2^ values and outperforms Bliss, Isserlis, and regression (see next section) (Fig 3, S1 Data). This formula includes only pair data (β = 0, α = 1/(M − 1)), and we hence name it the pairs model:

$$g^{pairs}{}_{1\ldots M}={\left(\prod g_{ij}\right)}^{1/(M-1)}$$ (2)

Thus, for triplets, the pairs model is the square root of the product of the 3 pair effects. One feature of the pairs model is that it is less sensitive to experimental noise than most other models in this class, because it uses only data for pairs; other models use both pair and single drug data, increasing the number of variables and hence the sensitivity to noise. Assuming independent multiplicative experimental noise for each measurement with standard deviation σ, the Bliss formula has total experimental noise of $\sqrt{M}\sigma$, the regression formula has larger noise of $\sigma \sqrt{\frac{M(M-1)}{2}+M{\left(M-2\right)}^{2}}$, and the pairs formula has noise of only $\sigma \sqrt{M/2(M-1)}$. For triplets (M = 3), for example, these noise terms are $\sqrt{3}=1.7$, $\sqrt{6}=2.5$, and $\sqrt{3/4}=0.9$ times σ for Bliss, regression, and pairs, respectively. The pairs model is expected to be most useful when data is noisy.

### The pairs formula improves on the Bliss approximation for high-order cocktail effects

**Fig 4:**
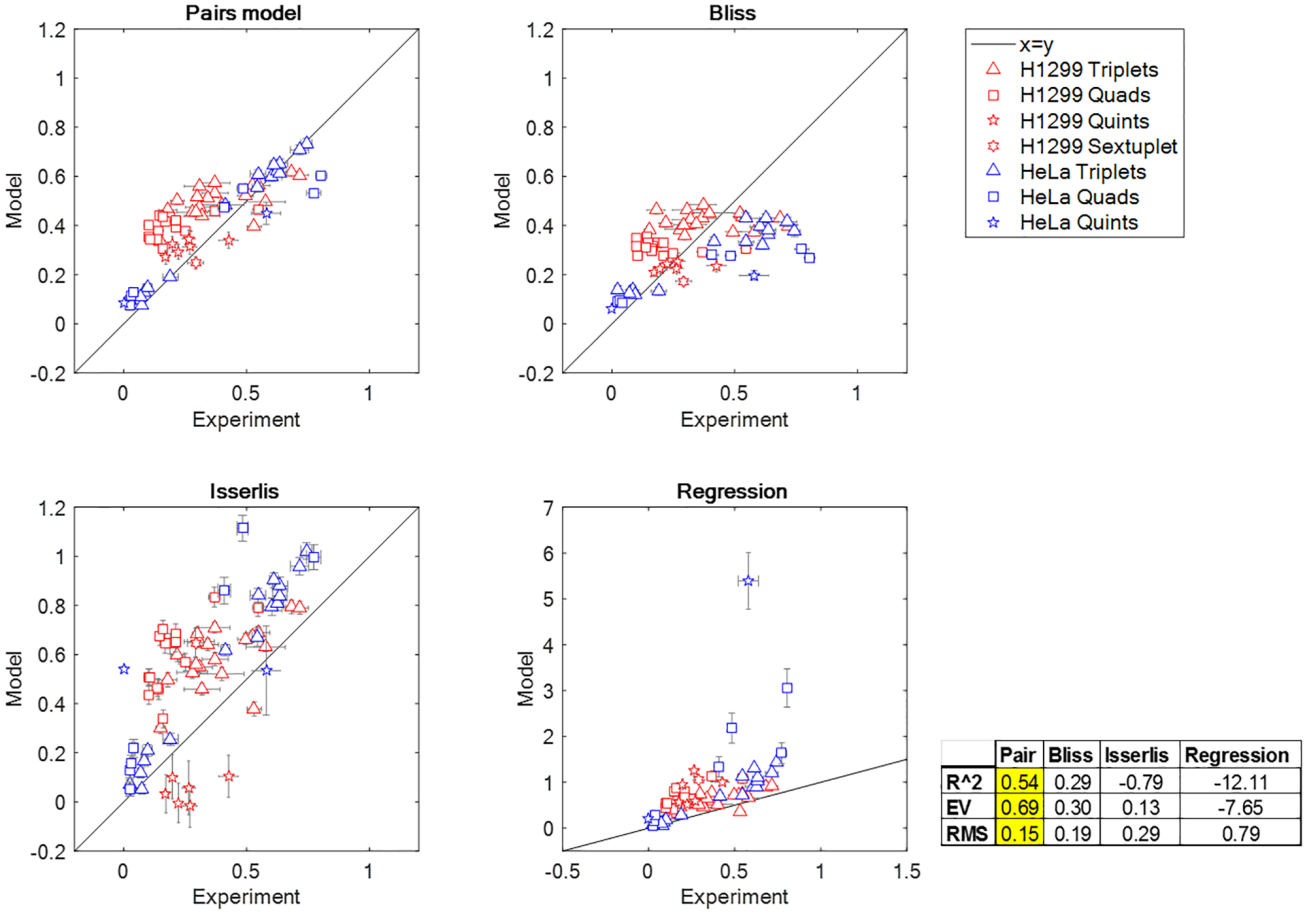
The pairs model improves on the Bliss approximation and other models for cancer drug cocktails. The plots compare the experimental data and the prediction of the indicated models.

We plot the agreement of different formulas to the present dataset in Fig 4 and S1 Data. The Isserlis and regression formulas are far from the data, as evidenced by their negative R^2^ values R^2^ = −0.79 and R^2^ = −12.1, respectively (negative R^2^ indicates lack of accuracy for the data mean since $R^{2}=1-\sum_{i}{\left(y_{i}-f_{i}\right)}^{2}/\sum_{i}{\left(y_{i}-{\bar{y}}_{i}\right)}^{2}$ for data y_i_ and prediction f_i_). Bliss independence has R^2^ = 0.29. The pairs model improves this to R^2^ = 0.54. This value of R^2^ is reasonable given the experimental noise in these measurements, which adds up when considering combinations of 4 to 6 drugs. Also, the higher the number of drugs, the more the potential for high-order effects above pairs, which cannot be captured by the present model.

We tested the pairs model also on previously published data for 1,360 antibiotic triplets and quadruplets by Wood et al. [28] The pairs model shows good fit to this data (R^2^ = 0.78), comparable to the Isserlis formula (R^2^ = 0.88). In this dataset, errors may be smaller than the present dataset (S3 and S5 Figs).

This antibiotic data are extensive enough to ask how well the models can rank the combinations in terms of efficacy. Efficacy ranking is of interest if one needs to prioritize potential cocktails based on measuring the pairs. We find that the pairs model shows 85% accuracy in identifying the top 10% most effective triplets (that is, triplets with lowest bacterial growth rate), compared to 75% accuracy in the Isserlis model, 22% for Bliss, and 10% for random (S6 Fig). The regression model shows worse accuracy than random.

The pairs model also describes the multidose cancer drug-triplet dataset by Zimmer et al. [29], with R^2^ = 0.7 compared to R^2^ = −0.29, −2.03, and 0.58 for Bliss, regression, and Isserlis formulae, respectively (S4 and S5 Figs). The model of [29] that uses multiple doses outperforms the pairs model (reaching R^2^ = 0.82).

## Discussion

We presented a fully factorial experiment on the effect of combinations of 6 chemotherapy drugs on viability of 2 cancer cell lines. The combinations showed varying degrees of synergy or antagonism. The synergy/antagonism of each combination was mostly consistent with that of the drug pairs that made up the combination. This led us to test models that predict combinations using only data on pairs and single drugs at a single dose per drug. We screened a class of log-linear models to develop a model for combinations based on pairs called the pairs model. In the pairs model, the effect of a combination of M drugs is equal to the product of the pair effects to the power 1/(M − 1). The pairs model improves on the predictions of the commonly used regression and Bliss models.

In addition to the 6 cancer drug combinations measured here, we also tested the pairs model on an additional 1,640 combinations form previous studies: 280 cocktails of 3 cancer drugs at 8 doses [29], 1,112 cocktails of 3 antibiotics, and 248 quadruplets of antibiotics [28]. The model predicts these combinations reasonably well. The model only works at the measured doses and is not able to predict effects of combinations at doses in which the pairs were not measured.

The synergy and antagonism of some of the combinations showed cell-line-specific effects; for example, the quadruplet CPT, CisPt, NCZ, and Etoposide is synergistic in H1299 but antagonistic in HeLa cells. A few combinations, however, showed synergy/antagonism that is consistent between the 2 cell lines tested. For instance, the pair MG132 and Etoposide is antagonistic in both cell lines, and the quadruplet CPT, CisPt, CbPt, and Etoposide is synergistic in both lines. If such cocktails turn out to be generally synergistic on a large number of cell lines and patient-derived samples, they may be a promising direction for therapy. The experimental dataset presented here is limited, and these findings must be tested on a broader number of cell lines and at multiple doses, as exemplified by the recent study by Horn et al. [5] on combinations of up to 6 drugs on multiple colorectal cancer cell lines.

The ability to use single and pair measurements to predict the effects of high-order combinations is a way to overcome the problem of combinatorial explosion. When measurements at several doses are available for pairs, the recent dose model of Zimmer and Katzir et al. provides excellent predictions for triplets and quadruplets of drugs as a function of dose [29]. That model uses multiple dose measurements to define response surfaces and therefore reduces the effect of measurement noise. In addition, the dose model provides interpolation of the effects of drug cocktails at different doses and therefore can be used to scan the combinatorial space of drug doses to find the most synergistic cocktails. The present study addresses the case where only a single dose is measured and suggests the pairs model as an improvement over Bliss independence and regression, especially in the presence of experimental noise. The pair model estimates the effect of cocktails based on the measured pairs, but it cannot provide estimates for doses for which the pairs were not measured. This single-dose scenario may be relevant to tests of drug combinations in large screens or on rare patient-derived material for personalized medicine applications [39–42].

## Materials and methods

### Cell lines

H1299 is a non-small-cell lung carcinoma cell line (clone 310806pl1H11 LMNA) described in Cohen et al. 2008. HeLa S3 cells were obtained from the American Type Culture Collection.

### Drugs

Drugs were treated as described in Geva-Zatorsky et al. 2011 [50]. Cisplatinum (P4394 Sigma) was dissolved in DMSO (hybri-max, D2650 Sigma), giving a stock solution of 25 mM; Nocodazole (M1404, Sigma) was dissolved in DMSO, giving a stock solution of 10 mM; Camptothecin (CPT, C9911 Sigma) and Etoposide (E1383 Sigma) were dissolved in DMSO, giving a stock solution of 0.3 mM; Carboplatin (C2538 Sigma) was dissolved in DDW, giving a stock solution of 500 mM; and MG132 (C2211 Sigma) was dissolved in DMSO, giving a stock solution of 0.32 mM. In each experiment, each drug was diluted to the desired concentration in transparent growth medium (RPMI 1640, 0.05% Penicillin-Streptomycin antibiotics, 10% FCS, with L-Glutamine, lacking riboflavin and phenol red, Bet Haemek, Biological Industries, catalog number 06-1100-26-1A). Normal transparent growth medium was replaced by the diluted drug solution [50].

### Viability assays

#### Neutral red

Cell viability was determined by neutral red assay (as described in Geva-Zatorsky et al. 2011 [50], Fluka Chemie, catalog number 72210, Buchs, Switzerland), (Zhang et al., 1990) as follows: Cells were plated in 96-well plates (Nunc, 164588) at 1.5 × 10^4^ cells per well. Cells were incubated at 37°C with 8% CO_2_ for 24 h in membrane-filtered growth media composed of RPMI 1640 with (+) L-Glutamine (GIBCO, catalog number 21875), 10% Fetal Calf Serum (certified fetal bovine serum, Biological Industries, catalog number 04-001-1A), and 0.05% Penicillin-Streptomycin antibiotics (Biological Industries, catalog number 03-031-1B). Next, cells were treated with the drugs and incubated for 48 h. Medium was then removed, and 100 μl neutral red reagent (diluted 1:100 in growth medium) was added to cells for a 1-h incubation. Neutral red buffer was washed, and 100 μl per well fixation buffer (1% CaCl2, 0.5% formaldehyde) was added. To extract the remaining cells, extraction buffer (1% glacial acetic acid, 50% ethanol) was added (100 μl per well). Cells were shaken for 20 s, and optical density (570 nm) was read by an ELISA reader. Following subtraction of blanks (wells with no cells), an average of replicates was calculated. Cell survival was determined as the percentage of the OD read in the untreated controls [50].

#### MTT assay

Cells were grown in 96-well plates as described above and were incubated with combinations of drugs for 48 h. Then, viability assay was done using the in vitro toxicology assay kit (MTT-based, TOX1 SIGMA). Cells were incubated with reconstituted MTT for 4 h, and absorbance was measured at 570 nm.

#### Computation of drug interaction

To determine the synergism/antagonism of a cocktail, we calculated I = log(1 + g_1..k_ − g^Bliss^_1..k_) for all biological repetitions of the drug cocktail g_1..k_ (the number of biological repeats ranged between 6 and 23). Then we used Matlab bootstrapping to calculate the mean of I and the 5/95 percentile confidence interval. If I > 0 and the 5th percentile > 0, we determine that the cocktail is antagonistic. If I < 0 and the 95th percentile < 0, we determine that the cocktail is synergistic. Otherwise, we determine that the cocktail is additive.

This interaction score is based on Bliss independence. We note that there are other ways to score synergy, which differ from Bliss in certain ways. First, Bliss does not account for dose additivity (for which Loewe models are most widely used), so a drug-with-itself combination could be scored as “synergy” or “antagonism.” Second, Bliss independence is a strong requirement for combinations, which may limit its utility as a synergy reference. Other scores include versions of HSA “best single agent”-referenced scores for absolute synergy and for differential synergy as described in Lehar et al. 2009 [6]. The absolute HSA synergy score typically shows a more balanced distribution of pair interactions. Differential synergy scores can avoid shifts towards positive absolute synergy at higher dimensions expected due to dose adding.

## Supporting information

S1 Fig Cell viability was measured using two different assays—MTT (blue line), and neutral red (red line), and showed similar results. **A)** Cell viability was measured in H1299 cell, 48h following MG132 drug treatment at several doses to determine LD20. **B)** Same as A) for Camptothecin (CPT).
(DOCX)

S2 Fig The distribution of drug interactions for the two cell lines. Ant denotes antagonism (I>0), Syn denotes synergism (I<0), and Add denotes additivity (I = 0 in the sense that zero lies in the 95% confidence interval of I).
(DOCX)

S3 Fig The pairs model shows a good fit for cocktails of antibiotics. The different plots show the comparison between the experimental data and the prediction of the indicated models. Experimental data from Wood et al [28].
(DOCX)

S4 Fig The pairs model shows the best fit among models for cocktails of three chemotherapy drugs on A549 cells. The different plots show the comparison between the experimental data and the prediction of the indicated models. Experimental data from Zimmer and Katzir et al [29].
(DOCX)

S5 Fig When scanning over to define different modes shows that the maximal R^2^ is received close to α = 1/(M-1) β = 0, which is the definition of the pairs model. The left panel is a comparison between the Pairs model, the Bliss model and the model defined by the α with the highest R^2^. The left panel is the R^2^ values of α = 0:0.05:1. The first row is for triplets of antibiotics- data taken from Wood et al. [28], the second row is cocktails of chemotherapy with A549 cells, data taken from Zimmer and Katzir et al. [29].
(DOCX)

S6 Fig The pairs model has the height rate of accuracy in ranking 10% of the most synergistic antibiotic triplets. We ranked the efficacy of 1112 cocktails of 3 antibiotics presented by wood et al 2012. We calculated the different models for this data, and ranked the models predictions. We compared the ranking of the experimental data and the ranks of the different model and calculated the rate of success. For 10% of the data (the enlarged image) the Pairs model show 85% rates of success, the Isserlis model shows 75%, the Bliss independence model show 22%, randomized data showed 10% success rate—as expected, and the regression model showed success rate which is lower than random. We randomized the data in two ways– 1) randomization over the pair measurements, calculating the Pairs model and ranking it (The green error bars). We did this procedure 1000 times and calculated the mean success rate and its std. 2) we randomized the ranks 1000 times and calculated the mean and std (light blue error bars).
(DOCX)

S1 Table Cell survival and interactions of all drug combinations in the present study. Viability was measured using the neutral read assay. 5%CI and 95%CI mark 5% and 95% confidence intervals from bootstrapping. Interaction is I = log(1+viability-Bliss) where Bliss is the product of the single drugs effects. Antagonism and synergy are marked in blue and pink, and denote cases where I>0 or I<0 together with the demand that I = 0 is outside the 95% confidence interval. Cocktails in bold are synergistic/antagonistic for both cell lines.
(XLSX)

S1 Data The data that was used to generate Figs 3 and 4. (XLSX)

S1 Code The code that was used to generate Fig 3. (M)

## Abbreviations

CbPt: Carboplatin
CisPt: Cisplatin
CPT: Camptothecin
Etopo: Etoposide
LD20: lethal dose 20%
NCZ: Nocodazole
