# Supplementary figures and images for: Prediction of drug cocktail effects when the number of measurements is limited
Source: PLoS Biol. 2017 Oct 26;15(10):e2002518. doi: 10.1371/journal.pbio.2002518 (PMC5675459; doi:10.1371/journal.pbio.2002518)

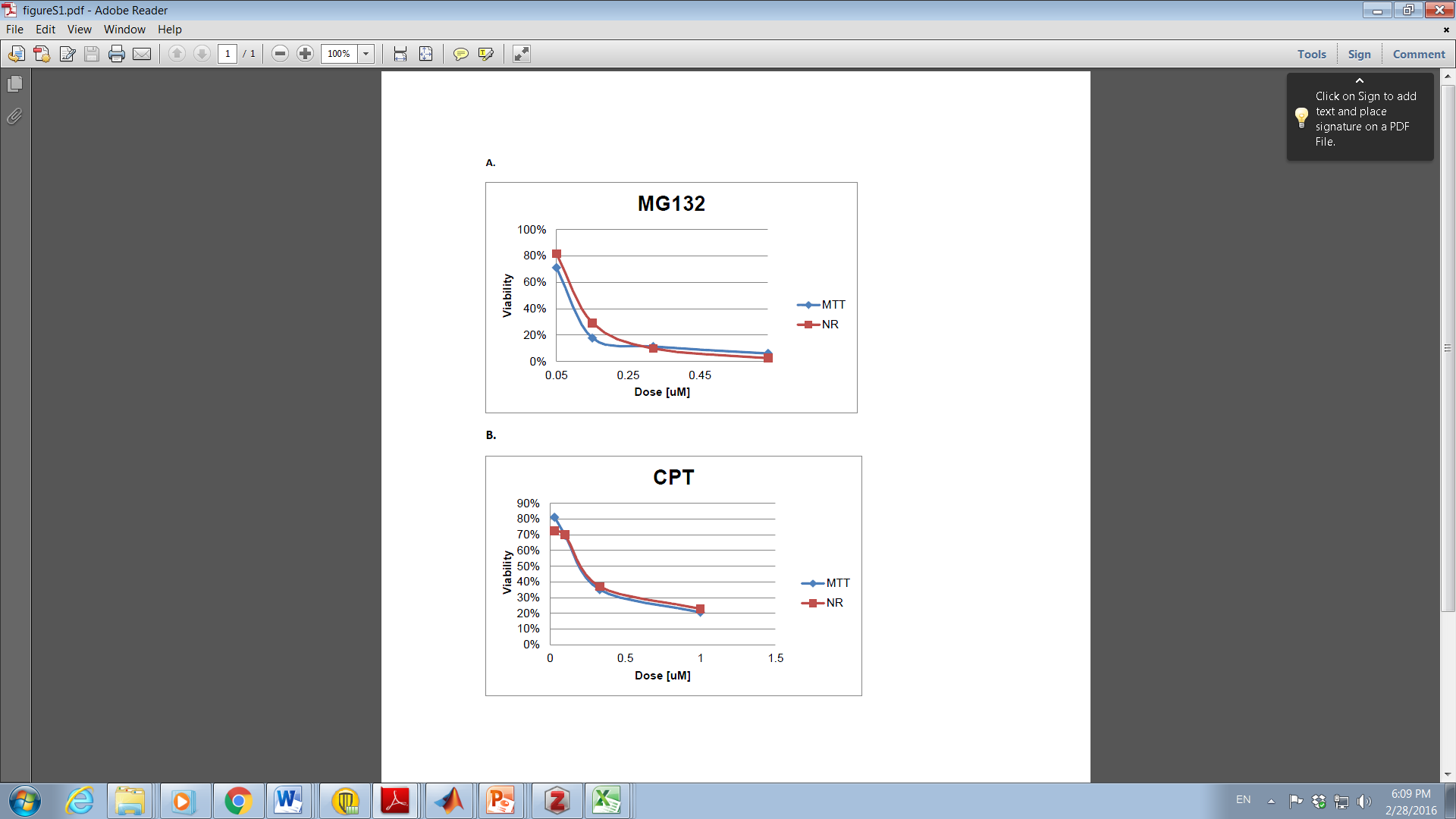

Supplement: S1 Fig — A) Cell viability was measured in H1299 cell, 48h following MG132 drug treatment at several doses to determine LD20. B) Same as A) for Camptothecin (CPT). (DOCX) [file pbio.2002518.s001.docx]

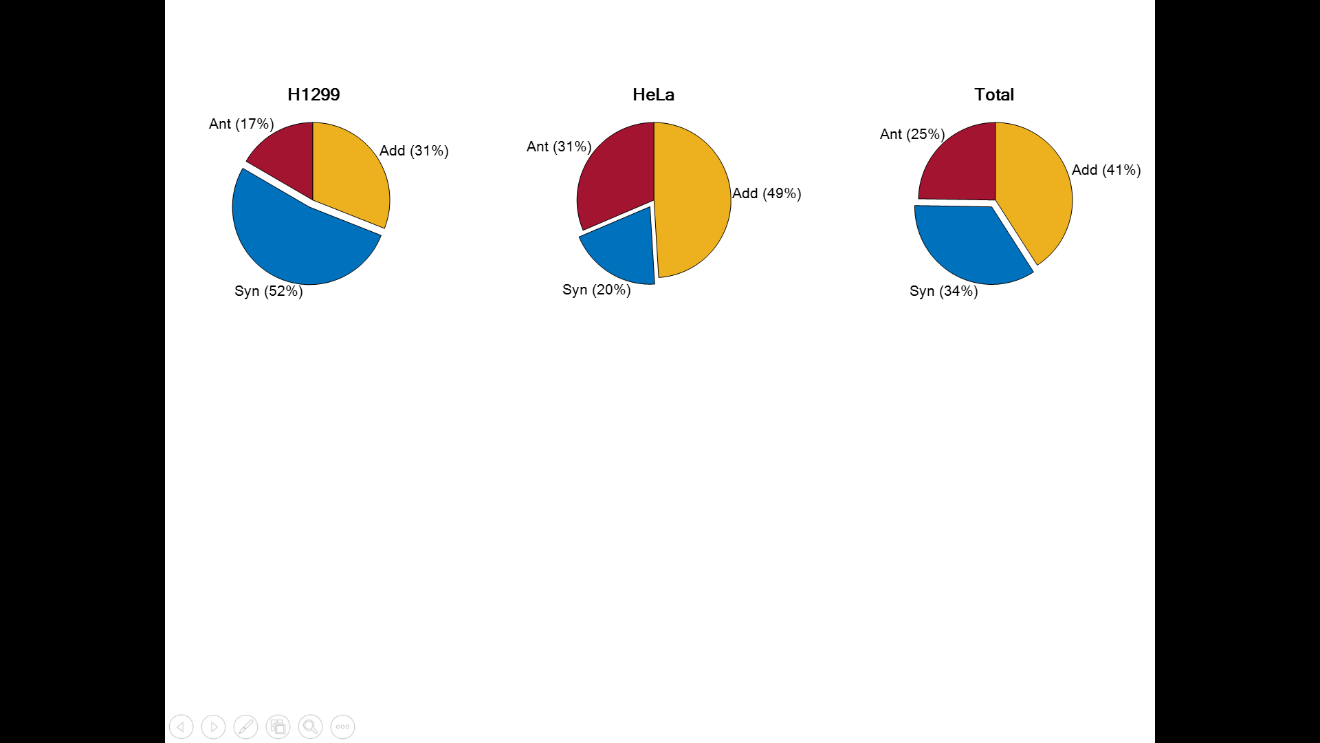

Supplement: S2 Fig — Ant denotes antagonism (I>0), Syn denotes synergism (I<0), and Add denotes additivity (I = 0 in the sense that zero lies in the 95% confidence interval of I). (DOCX) [file pbio.2002518.s002.docx]

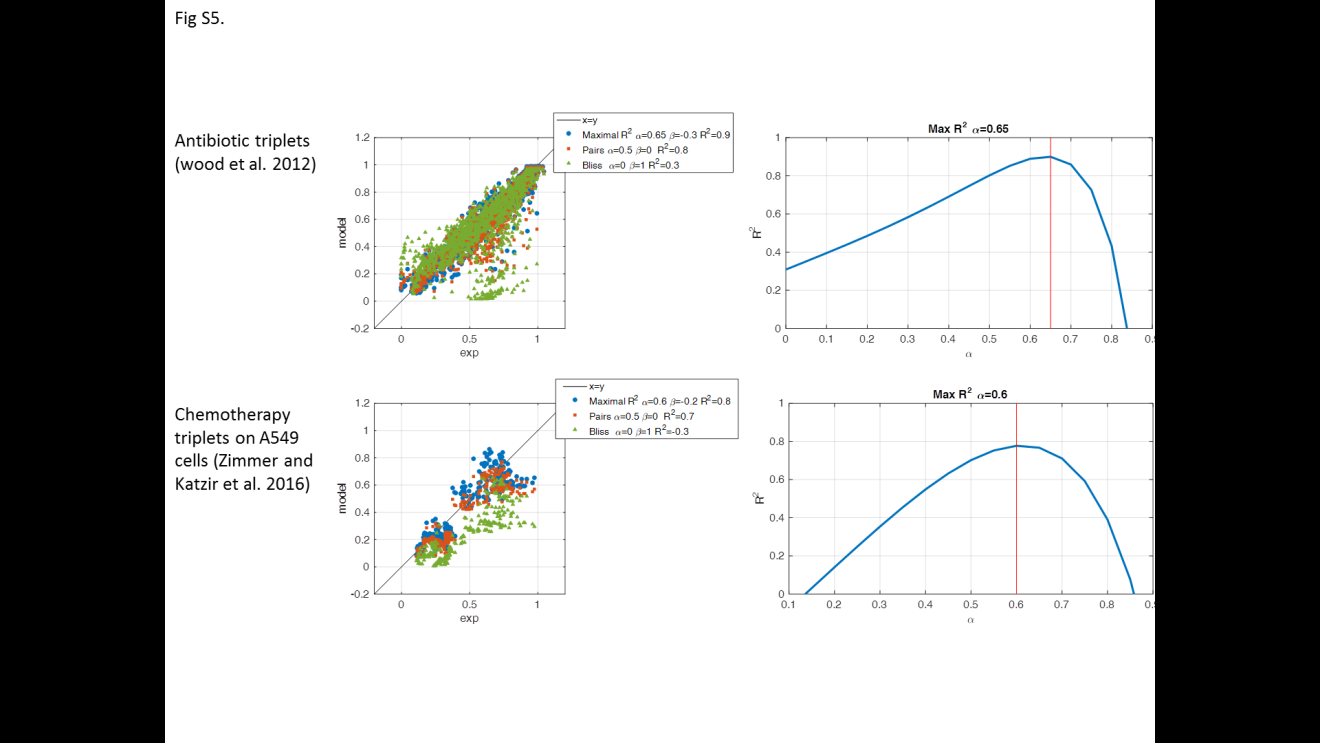

Supplement: S5 Fig — The left panel is a comparison between the Pairs model, the Bliss model and the model defined by the α with the highest R2. The left panel is the R2 values of α = 0:0.05:1. The first row is for triplets of antibiotics- data taken from Wood et al. [28], the second row is cocktails of chemotherapy with A549 cells, data taken from Zimmer and Katzir et al. [29]. (DOCX) [file pbio.2002518.s005.docx]
