# Supplementary material for: Prediction of drug cocktail effects when the number of measurements is limited
Source: PLoS Biol. 2017 Oct 26;15(10):e2002518. doi: 10.1371/journal.pbio.2002518 (PMC5675459; doi:10.1371/journal.pbio.2002518)
Supplement: Supplementary file 2 [file pbio.2002518.s003.docx]

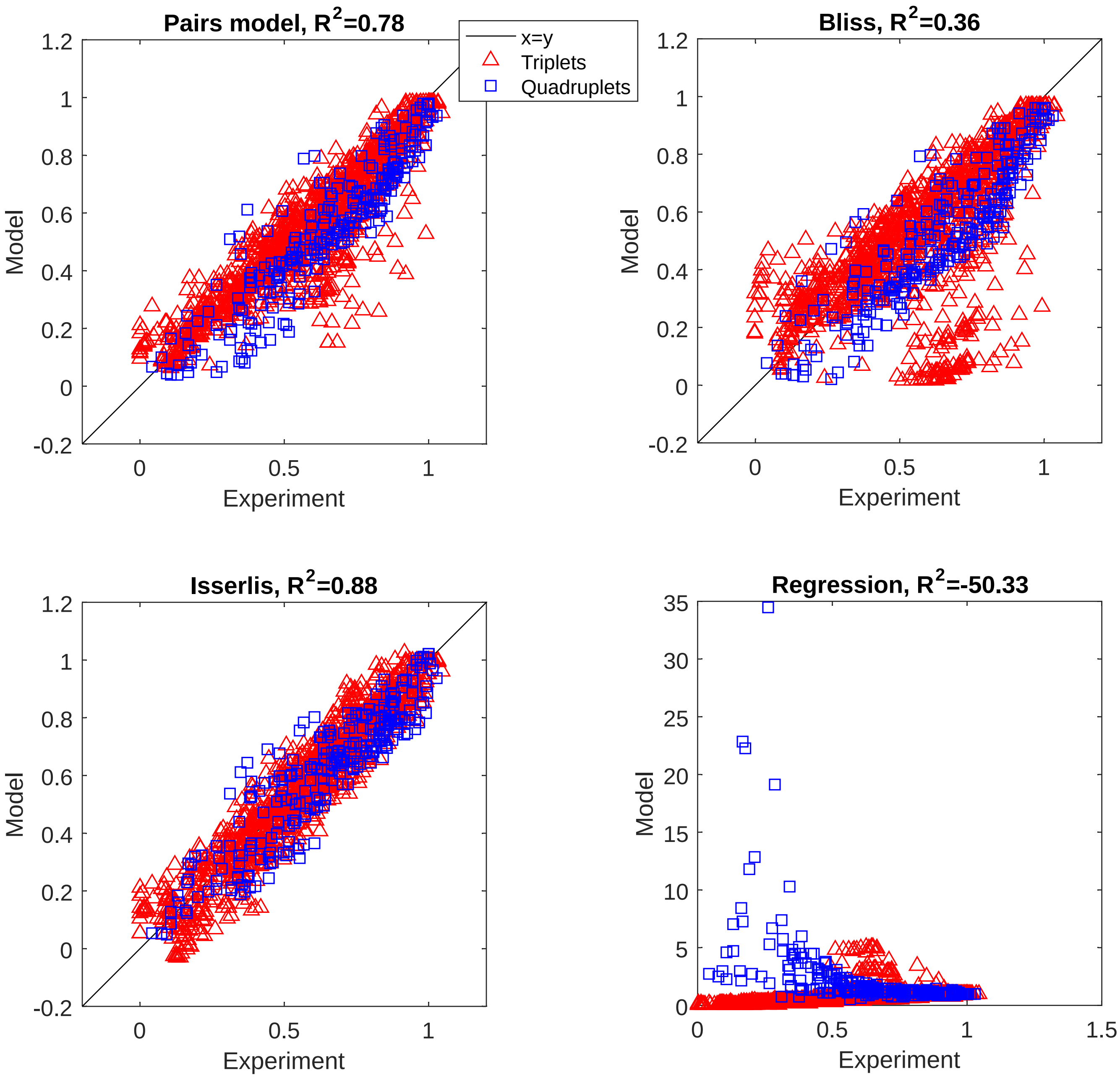

Pairs model, R^2=0.78
x=y
Triplets
Quadruplets
Bliss, R^2=0.36
Isserlis, R^2=0.88
Regression, R^2=-50.33
Model
Experiment
1.2
1
0.8
0.6
0.4
0.2
0
-0.2
0.5
35
30
25
20
15
10
5
1.5
