# Supplementary material for: Prediction of drug cocktail effects when the number of measurements is limited
Source: PLoS Biol. 2017 Oct 26;15(10):e2002518. doi: 10.1371/journal.pbio.2002518 (PMC5675459; doi:10.1371/journal.pbio.2002518)
Supplement: Supplementary file 3 [file pbio.2002518.s004.docx]

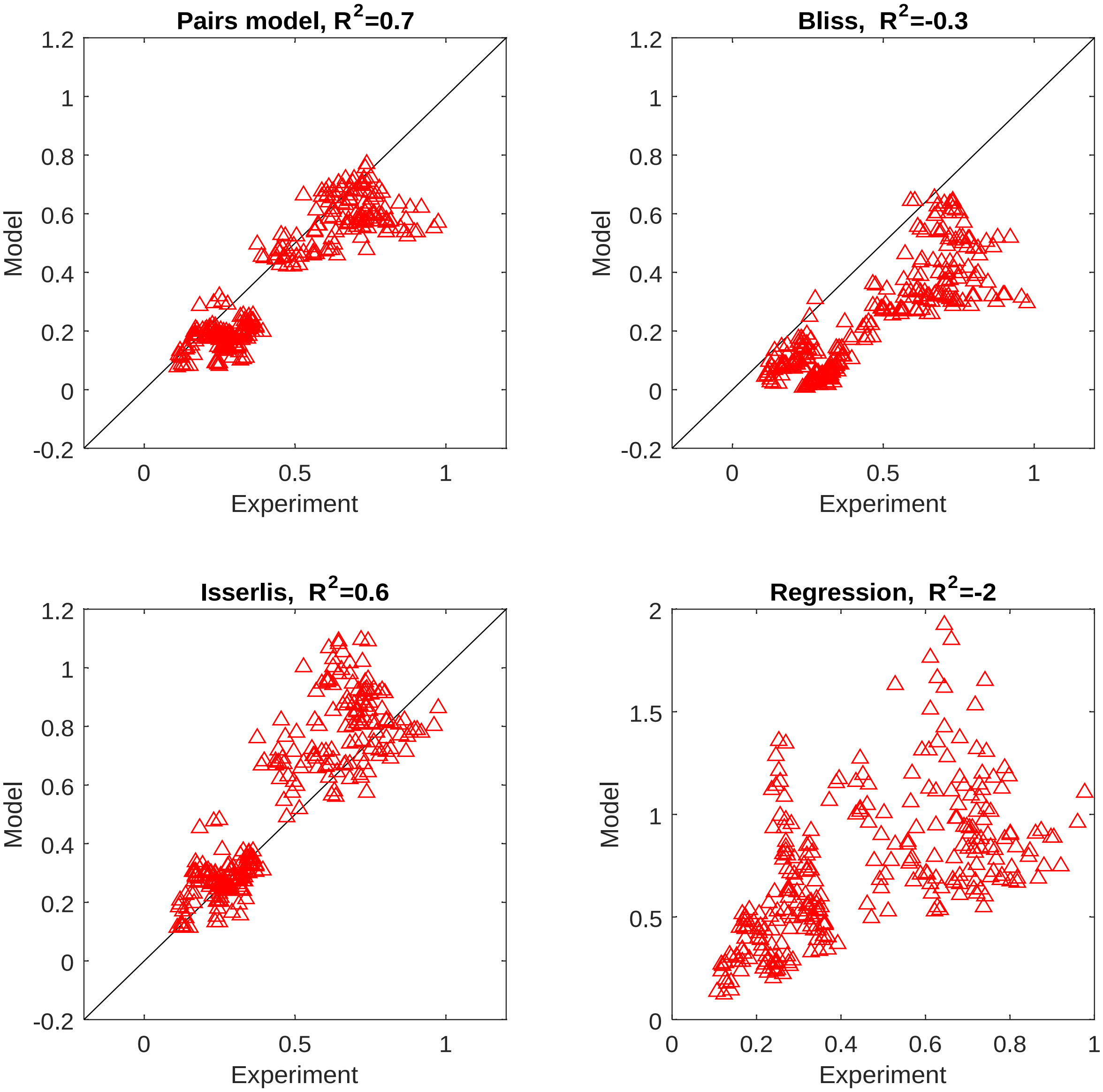
Pairs model, R^2=0.7
Bliss, R^2=-0.3
Isserlis, R^2=0.6
Regression, R^2=-2
Model
Experiment
1.2
1
0.8
0.6
0.4
0.2
0
-0.2
0
0.5
1
2
1.5
0.2
0.4
0.6
0.8
