# Supplementary material for: Prediction of drug cocktail effects when the number of measurements is limited
Source: PLoS Biol. 2017 Oct 26;15(10):e2002518. doi: 10.1371/journal.pbio.2002518 (PMC5675459; doi:10.1371/journal.pbio.2002518)
Supplement: Supplementary file 4 [file pbio.2002518.s006.docx]

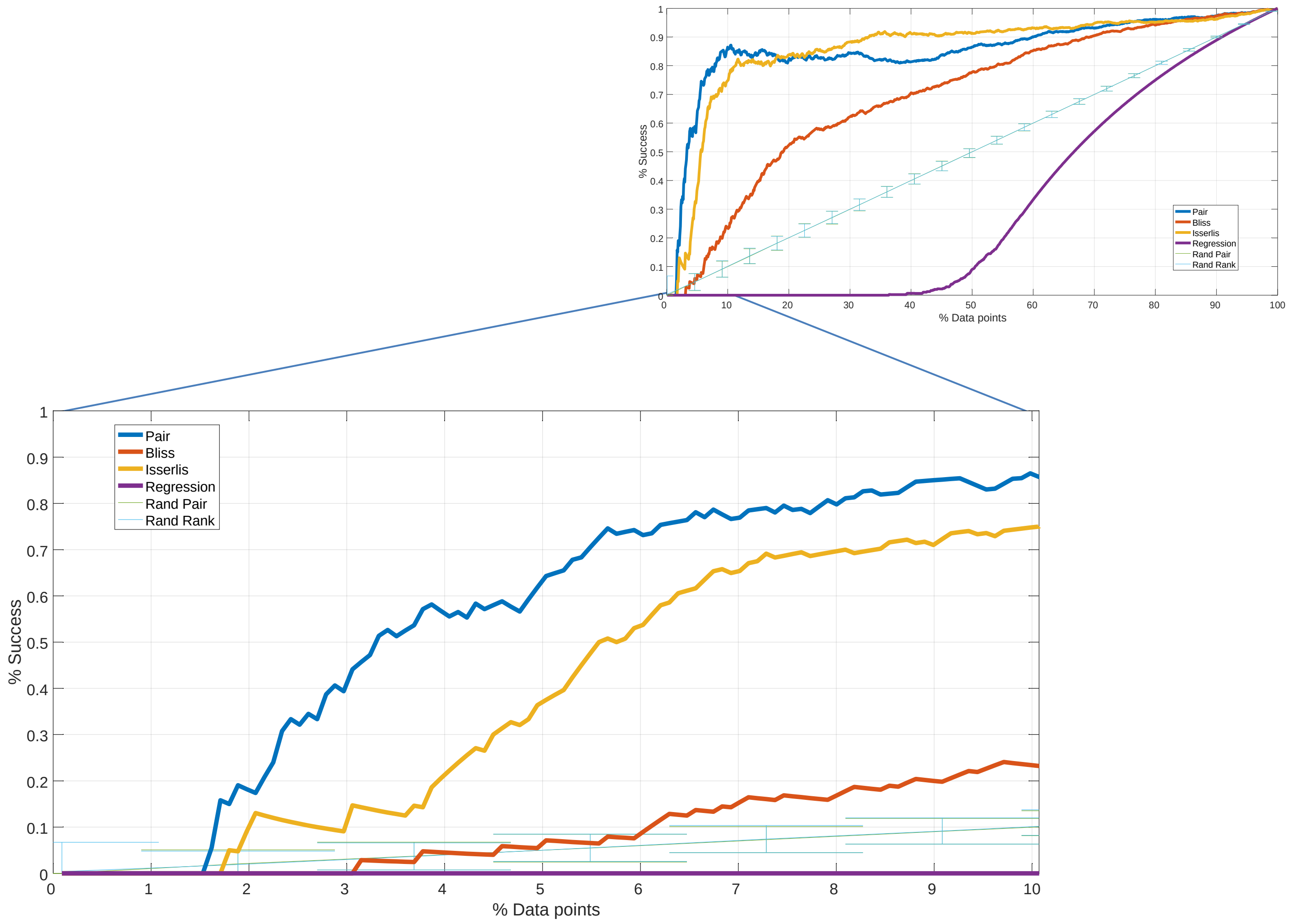

% Success
% Data points
Pair
Bliss
Isserlis
Regression
Rand Pair
Rand Rank
